# Ultra-Sensitive Fiber Refractive Index Sensor with Intensity Modulation and Self-Temperature Compensation

**DOI:** 10.3390/s19183820

**Published:** 2019-09-04

**Authors:** Zhaojun Li, Liangtao Hou, Lingling Ran, Jing Kang, Jiuru Yang

**Affiliations:** 1 College of Electronics Engineering, Heilongjiang University, Harbin 150080, China; 2 Key lab of Electronics Engineering, College of Heilongjiang Province, Heilongjiang University, Harbin 150080, China

**Keywords:** refractive index sensor, modal interferometer, intensity demodulation, temperature compensation

## Abstract

In this paper, a novel in-line modal interferometer for refractive index (RI) sensing is proposed and experimentally fabricated by cascading single-taper and multimode-double- cladding-multimode (MDM) fiber structure. Owing to evanescent field in taper area, the ultra-sensitive and linear intensity-responses to the varied surrounding RI are gained in both single- and double-pass structures. Moreover, the crosstalk from temperature can be effectively discriminated and compensated by means of the RI-free nature of MDM. The experimental results show that the RI sensitivities in single- and double-pass structures, respectively, reach 516.02 and 965.46 dB/RIU (RIU: refractive index unit), both with the slight wavelength shift (~0.2 nm). The temperature responses with respect to wavelength and intensity are 68.9 pm°C^−1^/0.103 dB°C^−1^ (single-pass structure) and 103 pm°C^−1^/0.082 dB·°C^−1^ (double-pass structure). So the calculated cross-sensitivity of intensity is constrained within 8.49 × 10^−5^ RIU/°C. In addition, our sensor presents high measurement-stability (~0.99) and low repeatability error (<4.8‰). On account of the ~620 μm size of taper, this compact sensor is cost-efficient, easy to fabricate, and very promising for the applications of biochemistry and biomedicine.

## 1. Introduction

Fiber refractive index (RI) sensors have been widely applied in biology, chemistry, medicine, and other fields with the merits of high sensitivity, compact size, and low-cost [1]. Recently, massive RI sensors include fiber Bragg grating (FBG) sensors [2,3], long-period fiber grating sensors [4,5], surface plasmon resonance refractometers [6,7,8], photonic crystal fiber refractometers [9,10,11], microfiber/microfiber coupler [12,13,14,15], and model interferometer [16,17,18,19,20,21] have been extensively investigated and the sensitivity record is continuously updated. So far, to the best knowledge of the authors, wavelength sensitivity as high as ~2 × 10^5^ nm/RIU (RIU: refractive index unit) is achieved in a polarization-maintaining microfiber based structure [22]. Nevertheless, it is worth noting that wavelength modulated schemes surely require the support of expensive optical spectrum analyzer (OSA) to monitor spectral shift.

Comparatively, intensity modulation based RI sensors are more practical but it is rarely reported that they can be implemented by a cost-effective power meter [18,23]. Zhou et al. proposes an offset-core thin-core fiber (TCF) based structure and the sensitivity of −202.46 dB/RIU is obtained [24]. To enhance sensitivity, lots of schemes based on up- and down-taper are frequently proposed due to the extensive loss of the evanescent field [25,26,27]. Moreover, based on the RI-free feature of multimode fiber (MMF), the bias-taper based structures with self-temperature compensation are respectively fabricated by arc-discharge and flame-brushing techniques. The reported sensitivities are about −340 dB/RIU with the linearity of >0.98 [28,29]. Further, Chen et al. form a weak Fabry–Perot cavity by the slight RI difference of TCF and single mode fiber (SMF) and an exceeding −1100 dB/RIU sensitivity is demonstrated, but only at the point of 1.4305 [30]. To overcome this limitation, Shi et al. insert a no-core fiber (NCF) into a fiber ring-cavity laser with FBG and its output power is only sensitive to the variation of external RI. The segmented intensity sensitivities with low temperature crosstalk are presented in [31], which are 196.1 dB/RIU (in the range of 1.335–1.354) and 744.6 dB/RIU (in the range of 1.354 to 1.367), respectively.

In this paper, we fabricate a novel in-line modal interferometer for RI sensing through cascading the single-taper and multimode-double-cladding-multimode (TMDM) fiber structure. In the proposed TMDM, the taper area is intensity sensitive to the varied surrounding RI (SRI) owing to the evanescent field, and the part of MDM just serves to monitor the change of ambient temperature because of its RI-free nature. Comprehensive RI measurements are performed in terms of sensitivity, stability, and repeatability. The experimental results show that the sensitivity up to 965.46 dB/RIU is gained in double-pass structure with the ~0.2 nm wavelength shift. Moreover, the intensity drift of temperature is constrained within ~0.1 dB/°C and the calculated cross-sensitivity is about 8.49 × 10^−5^ RIU/°C. Additionally, the proposed sensor with ultra-high sensitivity and a narrow refractive index range can accurately detect specific biological, medical, or chemical agents. It has the merits of compactness, cost-efficiency, and ease of fabrication, which is very potential to be a minimized biochemical sensor.

## 2. Principle and Fabrication

**Figure 1:**
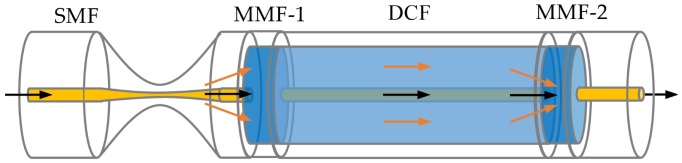
The schematic diagram of the single-taper and multimode-double-cladding-multimode (TMDM) with a single-pass structure.

The TMDM with a single-pass (SP) structure is illustrated in Figure 1, which includes two short-length MMFs (denoted by MMF-1 and MMF-2, respectively), a piece of tapered SMF, and a section of double-cladding fiber (DCF, SM-9/105/125-20A, Nufern, Hartford, CT, USA). In particular, the fiber-core and two fiber-claddings (inner-cladding and outer-cladding) diameters of the adopted DCF are 9, 105, and 125 μm, respectively. The matched MMFs are chosen with the fiber-core and fiber-cladding diameters of 105 and 125 μm. Therefore, the incident light from the taper area is split by MMF-1 with the ratio of *κ*_1_ and propagates in the fiber-core and fiber-cladding. The guide mode and excited cladding modes are recoupled by MMF-2 with the ratio *κ*_2_. Because the phase delay (denoted by Δ*ϕ*) caused by the RI difference of the fiber-core mode and fiber-cladding mode, a stable in-line Mach–Zehnder interferometer (MZI) is formed. It is well known that its transmitted intensity can be described by

(1) $$I=I_{co}+I_{cl}+2\sqrt{I_{co}I_{cl}}\cos(\Delta \varphi )$$

where *I_co_* and *I_cl_* are the intensities of the fiber-core and fiber-cladding modes, respectively. Δ*ϕ* can be written as

(2) $$\Delta \varphi =\frac{2\pi }{\lambda }\left(n_{co}-n_{cl}\right)\cdot L=\frac{2\pi \;\Delta n_{eff}\;L}{\lambda }$$

where *n_co_*, *n_cl_*, and Δ*n_eff_* are the effective RI of the fiber-core mode, the effective RI of fiber-cladding mode, and the effective RI difference of the fiber-core and fiber-cladding modes, respectively. *L* and *λ* are the DCF length and the incident light wavelength, respectively. When the condition Δ*ϕ* = (2*m* + 1)*π*, the interference dip wavelength (*λ_dip_*) of the spectrum will be

(3) $$\lambda_{dip}=\frac{2\Delta n_{eff}L}{2m+1}$$

where *m* is an integer. Note that, the modal interference generates just between the fiber-core mode and the inner-cladding mode because of the limitation of MMF-1. In addition, the incident light will extensively leak into the cladding and air in taper area due to the evanescent field. Moreover, this loss will increase with the rise of SRI according to [32]. This means the introduced single-taper can be used as an attenuator related to SRI.

Here we define the loss factor as *α*_(*n*)_ and assume *κ*_1_ = *κ*_2_ = *κ*, Equation (1) is then modified by

(4) $$\begin{array}{l} I=I_{co}+\alpha_{\left(n\right)}\kappa_{1}\kappa_{2}I_{co}+2\sqrt{I_{co}\alpha_{\left(n\right)}\kappa_{1}\kappa_{2}I_{co}}\cos(\Delta \varphi )\\ \;=(\alpha_{\left(n\right)}\kappa^{2}+1)I_{co}+2\kappa \;I_{co}\sqrt{\alpha_{\left(n\right)}}\;\cos(\Delta \varphi ) \end{array}$$

Furthermore, the normalized fringe visibility is defined as:

(5) $$V=\frac{2\kappa \;I_{co}\sqrt{\alpha_{\left(n\right)}}}{(\alpha_{\left(n\right)}\kappa^{2}+1)I_{co}}=\frac{2\kappa \sqrt{\alpha_{\left(n\right)}}}{\alpha_{\left(n\right)}\kappa^{2}+1}$$

Equation (5) shows that, for the given *κ* the value of *V* is proportional to $\sqrt{\alpha_{\left(n\right)}}$ and that means an RI sensing test with intensity modulation can be achieved by the proposed SP structure.

**Figure 2:**
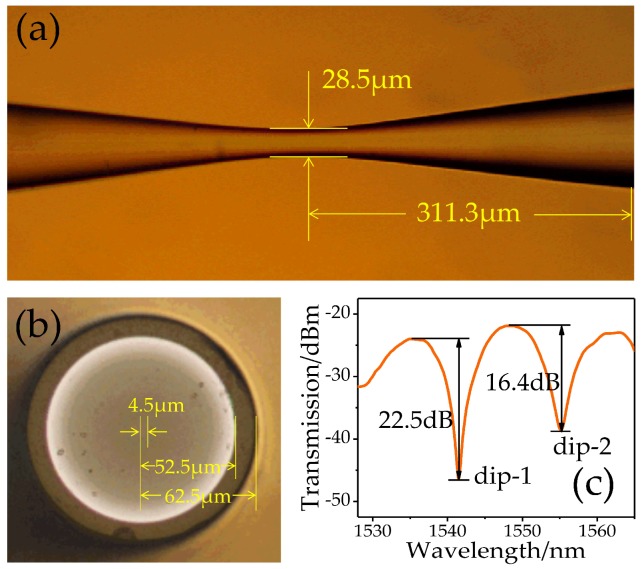
(**a**) Microscope image of the fabricated taper. (**b**) The cross-sectional morphology of double-cladding fiber (DCF). (**c**) The transmission spectrum of the single-pass (SP) structure.

In fabrication, both ends of a 45-mm long DCF are firstly spliced with two sections of MMF (MM-S105/125-22A, Nufern, Hartford, CT, USA) by a commercial fusion splicer (FSM-100P, Fujikura, Tokyo, Japan) and keep the length of MMF are about 0.4 mm by cutting to avoid the possible multimode interference. Then the formed MDM structure is respectively spliced with two pieces of SMF (Corning SMF-28) as the lead-in and lead-out fibers. Finally, an adiabatic taper locating at the middle of lead-in SMF is completed by the arc-discharge technique. In detail, the power of pre-discharge and main-discharge are 30 bits and 80 bits, and the corresponding discharge times are 150 and 2200 ms, respectively. The waiting time and the speed of welding are 1200 ms and 0.15 μm/ms, respectively. From Figure 2a, the symmetric transitions are demonstrated with the length of ~311.3 μm and the waist-diameter is *d_w_* = 28.5 μm. Further, the cross-sectional morphology of DCF and the transmission spectrum of the SP structure (in air) are also given in Figure 2b,c. Obviously, there are two fringes, respectively, located at 1541 and 1555 nm (denoted by dip-1 and dip-2) with the visibilities of 22.5 and 16.4 dB. In addition, the interval spacing between dip-1 and dip-2 is about 14 nm.

## 3. Experiments and Results

**Figure 3:**
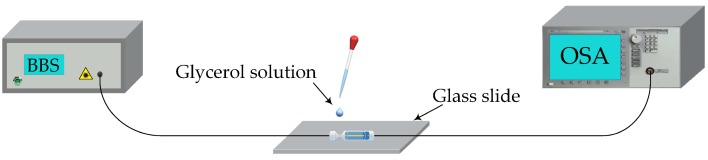
Experimental setup.

The experimental setup is shown in Figure 3. The fixed sensor head is connected to a broadband source (BBS, homemade, working in 1520–1565 nm) and an OSA (Agilent 86142 B, resolution: 0.06 nm/0.01 dB, Palo Alto, CA, USA). We prepare glycerol solutions with different concentrations and then perform a comprehensive RI test at room temperature of 24 ± 0.5 °C. Note that, the RI of glycerol solution is collimated using an Abbe refractometer before each test. We drip the glycerin solution on the sensor head and record the spectrum. The sensor head is thoroughly cleaned with anhydrous ethyl alcohol after each recording of the spectrum.

**Figure 4:**
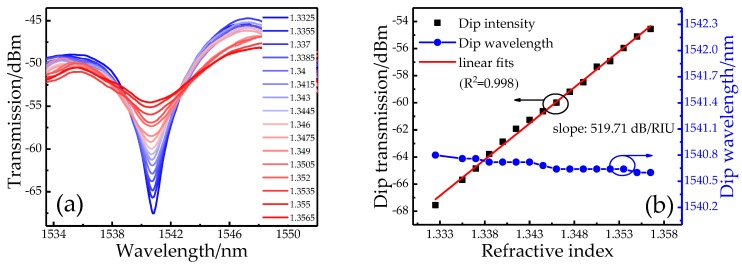
(**a**) Transmission spectra of SP structure and (**b**) the wavelength and intensity responses with a varied surrounding refractive index (SRI).

**Figure 5:**
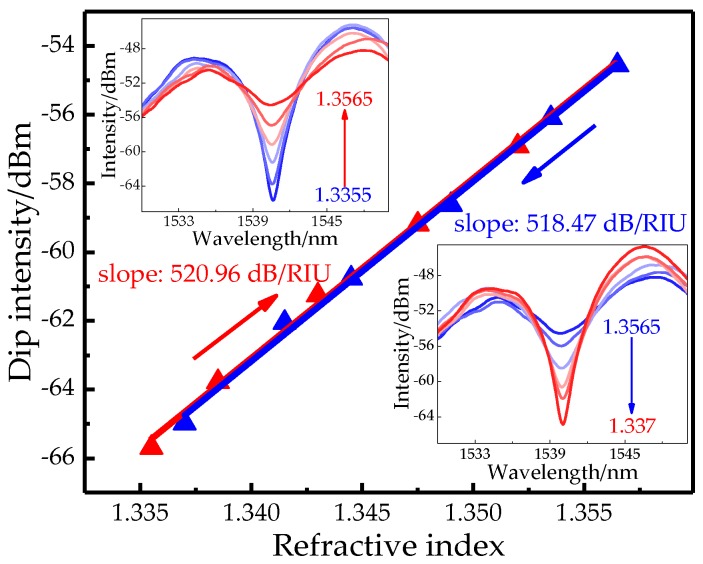
Intensity responses with the rising and reducing of SRI. Insets: transmission spectra.

Here dip-1 is selected to monitor the variation of SRI due to its larger visibility. From Figure 4a, the intensity of dip-1 is quickly increased with the added solution concentration. By calculation the total increment reaches 12.988 dB (from −67.544 to −54.556 dB) in the range of 1.3325~1.3565 RIU. Figure 4b presents an intensity response of 519.71 dB/RIU is obtained with high linearity. On account of 0.01-dB resolution, the detection limit of SP structure is 1.92 × 10^−5^ RIU. Comparatively, dip-1 merely shifts about −0.2 nm (from 1540.8 to 1540.6 nm). Since the isolation of the outer cladding of the DCF makes the DCF region insensitive to the SRI, and the energy loss of the taper increases as the SRI increases, only the power in the received spectrum is affected by an RI change without the wavelength shift. In addition, the repeatability test is conducted and the solution concentration is increased (decreased) by adding the ratio of glycerol (distilled water). As shown in the insets of Figure 5, the intensity variations of fringe visibility present a high consistence for the increased and decreased concentrations of glycerol solution. In the range of 1.335–1.358, the intensity sensitivities of 520.96 and 518.47 dB/RIU with high linearity (>0.99) are gained for the rising and reducing of SRI, respectively. By calculation the repeatability error of our sensor is ~4.8‰.

**Figure 6:**
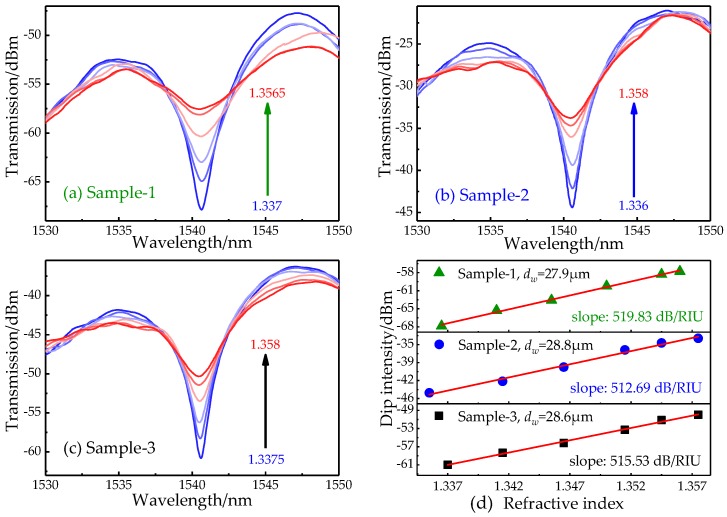
Stability measurements. (**a**–**c**) The transmission spectra and (**d**) intensity responses with varied SRI of three new SP-based samples.

Furthermore, to verify stability, three new SP-based samples are prepared with the same DCF length (~45 mm) and similar waist-diameters (*d_w_* = 27.9, 28.8, and 28.6 μm, respectively). The transmission spectra of three new SP-based samples are shown in Figure 6a–c. From Figure 6d, the intensities of three dips consistently increased with the rise of SRI, and the sensitivities are 519.83, 512.69, and 515.53 dB/RIU with the linearity of 0.99. By calculation, mean sensitivity (*S_m_*) is $\frac{1}{3}\sum_{i}S_{i}=516.02$ dB/RIU, and the stability is equal to $\frac{1}{3}\sum_{i}1-\frac{|S_{i}-S_{m}|}{S_{m}}=0.995,\;i=1,2,3$, where *S_i_* is the sensitivity of Sample-*i*. The standard deviation (SD) is equal to $\sqrt{\frac{1}{3}\sum_{i=1}^{3}{\left(S_{i}-S_{m}\right)}^{2}}=2.87$ dB/RIU, and the standard error is equal to $\frac{SD}{\sqrt{3}}=1.657$ dB/RIU. The slopes of all samples are distributed in the range of (*S_m_* − 3σ, *S_m_* + 3σ) and the proposed sensor presents a high stability. Therefore, the mean resolution of SP structure is 1.938 × 10^−5^ RIU.

**Figure 7:**
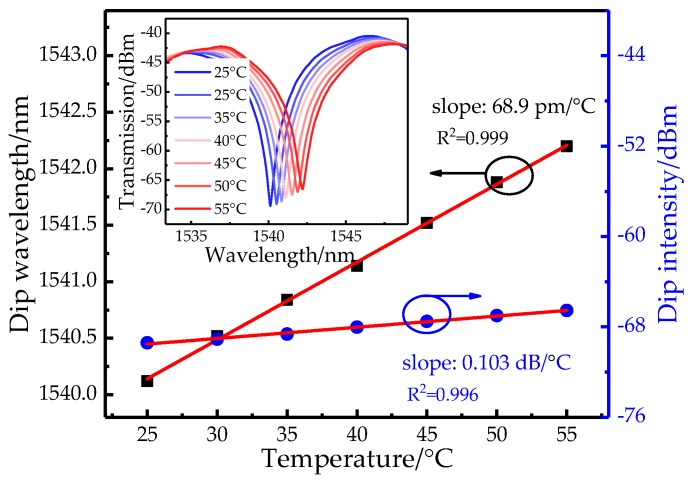
The wavelength and intensity responses with varied temperature. Inset: transmission spectra of SP structure.

In order to quantify crosstalk, the temperature response is also investigated by placing the sensor head into an electric thermostat. The inset of Figure 7 shows that the fringe dip has a clear red-shift as the temperature increasing from 25 °C to 55 °C. From Figure 7, a linear relation is found and the sensitivity is ~68.9 pm/°C. Comparatively, there is a slight increase for the fringe intensity when the temperature is increased and the calculated sensitivity is ~0.103 dB/°C. So the intensity error caused by temperature cross-sensitivity is 1.98 × 10^−4^ RIU/°C when non-temperature-compensation is employed.

**Figure 8:**
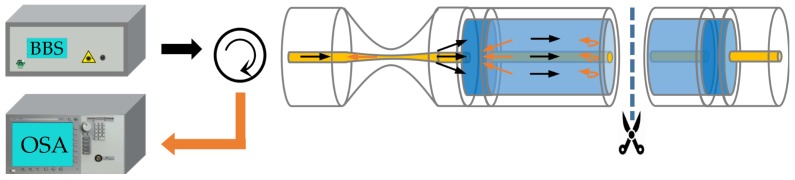
Schematic and experimental setup of TMDM with a double-pass structure.

Furthermore, to further enhance sensitivity, the sensing characteristics of TMDM with double-pass (DP) structure are experimentally performed. As shown in Figure 8, in DP structure, the light beams will be reflected by a well-cut end-face of DCF and pass through the taper area again. This surely brings a twice-loss and leads a doubled sensitivity improvement. A new DP-based sample is fabricated with a shorter DCF length (~23.5mm) and a similar waist-diameter (*d_w_* = 28.8 μm) for discrimination.

**Figure 9:**
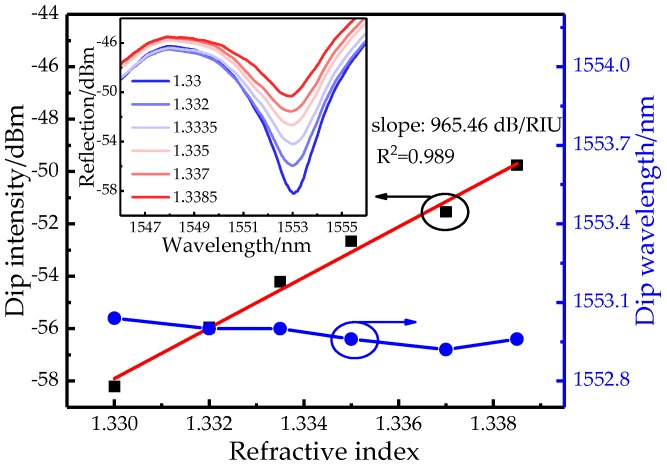
Wavelength and intensity relationships in a double-pass (DP) structure with varied SRI.

**Figure 10:**
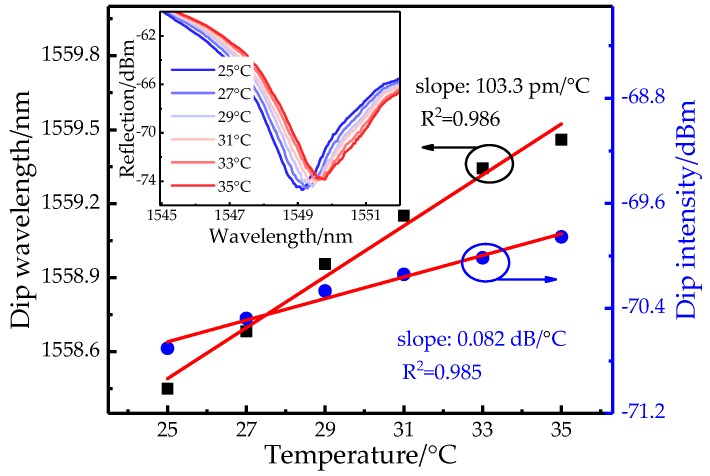
Wavelength and intensity relationships in DP structure with varied temperature.

The DP structure is connected with BBS and OSA by a circulator and its RI response is measured and presented in Figure 9. The inset shows that the DP structure has a much quicker intensity increase when the SRI is rising but with a ~8.5-dB reduction of visibility maybe due to the loss of the fiber-core mode at the reflected end-face. In detail, the total increment is 8.455 dB in the range of 1.33~1.339 RIU and the corresponding intensity sensitivity reaches 965.46dB/RIU with a linearity of 0.989. Moreover, the maximum value of wavelength drift is merely ~0.12 nm. Thus, approximately 1.85-time enhancement of detection limit (1.036 × 10^−5^ RIU) is gained in the DP structure. Note that the actual sensitivity is not fully doubled possibly due to the fabricated taper difference in terms of length and waist-diameter [26]. In addition, the measurement range of the proposed sensor increases as the fringe visibility increases, and the fringe visibility is related to the waist-diameter and length of the taper. Therefore, the measurement range can be expanded by appropriately adjusting the waist-diameter and length of the taper. For comparison, the temperature response of DP structure is also measured and demonstrated in Figure 10. The inset shows a clear red-shift with the added temperature but the increased intensity is merely 0.849 dB in the range of 25~35 °C. The temperature sensitivity with respects to wavelength and intensity are 103.3 pm/°C and 0.082 dB/°C with a linearity of 0.985. So the intensity crosstalk from the temperature is further restrained within 8.49 × 10^−5^ RIU/°C in the DP structure.

Further, the variations of RI and temperature in DP structure can be discriminated by the inversion matrix method [28], which can be described as

(6) $$\left[\begin{array}{l} \Delta T\\ \Delta n \end{array}\right]=\frac{1}{D}\left[\begin{array}{l} \;k_{In}\;-k_{\lambda n}\\ -k_{IT}\;k_{\lambda T} \end{array}\right]\;\left[\begin{array}{l} \Delta \lambda \\ \Delta I \end{array}\right],$$

where ∆*n* and ∆*T* are the variations of RI and temperature, respectively. ∆*λ* and ∆*I* are the wavelength shift and intensity change. *D* = *k_λT_k_In_ − k_IT_k_λn_*, where *k_λT_* = 0.103, *k_λn_* = 0 are wavelength sensitivities of temperature and RI in the DP structure, *k_In_* = 965.46, *k_IT_* = 0.082 are intensity sensitivities of RI and temperature in the DP structure. Consequently, the matrix will be changed as

(7) $$\left[\begin{array}{l} \Delta T\\ \Delta n \end{array}\right]=\frac{1}{99.44}\left[\begin{array}{l} 965.46\;0\\ -0.082\;0.103 \end{array}\right]\;\left[\begin{array}{l} \Delta \lambda \\ \Delta I \end{array}\right]$$

and the measurement of RI without the crosstalk of temperature can be completed.

Table 1 compares the various fiber RI sensors with our schemes (Note, the resolution of receivers in calculation are the same with 0.06 nm/0.01 dB). It is obvious that the competitive sensitivities are presented in both SP and DP structures. A near 1 × 10^3^ dB/RIU is gained by the DP structure and is only slightly lower than the result reported in [30] but it has a better linearity (0.989) in the range from 1.33 to 1.339 RIU. Besides, on account of the self-temperature compensation and ultra-small size (~620 μm), our schemes have potential and are suitable to be integrated and applied in biochemical fields, such as lab-on-chip.

## 4. Conclusions

In conclusion, a compact RI sensor is experimentally fabricated and demonstrated by cascading the tapered SMF and multimode-double-cladding-multimode fiber structure and the performance of SP and DP based structures are measured and compared in terms of RI and temperature responses. Experimental results show that only the taper area (~620 μm) is sensitive to the varied SRI by an intensity modulation. Owing to the introduced twice-loss, the sensitivity of near 1 × 10^3^ dB/RIU is gained in the DP structure with a high linearity (>0.989) and low wavelength-shift. The corresponding detection resolution is 1.036 × 10^−5^ RIU and by the inversion matrix method, the measurement of RI without the crosstalk of temperature can be completed. More importantly, the proposed sensor presents a high practicality in terms of repeatability and stability. Therefore, such a compact and stable sensor with self-temperature compensation and ultrahigh linear sensitivity is very promising in high-resolution biochemical sensing.

## Figures and Tables

**Table 1:** Comparisons of various fiber RI sensors.

Structures	Sensitivity	Resolution (RIU)	Linearity	RI Range (RIU)	Refs
Tilted FBG	−574.23 nm/RIU	1.045 × 10^−4^	0.999	1.40–1.45	[3]
Long-period grating	505 nm/RIU	1.188 × 10^−4^	/	1.333–1.354	[5]
Microfiber coupler	12,020 nm/RIU	4.99 × 10^−6^	0.996	1.3333–1.3341	[13]
S-tapered fiber	268.8 nm/RIU	2.23 × 10^−4^	0.982	1.332–1.387	[20]
Eccentric hole-assisted dual-core fiber	102.2 dB/RIU	9.785 × 10^−5^	0.981	1.335–1.37	[23]
Offset-core TCF	−202.46 dB/RIU	4.939 × 10^−5^	/	1.42	[24]
Tapered fiber tip with air bubble	442.59 dB/RIU	2.259 × 10^−5^	0.994	1.333–1.38	[25]
Bias-tapered MMF	345.78 dB/RIU	2.892 × 10^−5^	0.998	1.336–1.351	[29]
Weak-FP based TCF	240 dB/RIU	4.167 × 10^−5^	/	1.3326–1.4305	[30]
	1110.7 dB/RIU	9.003 × 10^−6^	/	1.4305	
NCF-based laser sensor	−196.1 dB/RIU	5.099 × 10^−5^	0.997	1.335–1.354	[31]
	−744.6 dB/RIU	1.343 × 10^−5^	0.997	1.354–1.367	
SP-TMDM	516.02 dB/RIU	1.938 × 10^−5^	0.998	1.33–1.356	Our works
DP-TMDM	965.46 dB/RIU	1.036 × 10^−5^	0.989	1.33–1.339	
